# Trends in Postpartum Depression and Anxiety Diagnoses Among U.S. Active Component Service Women, 2010–2024

**Published:** 2026-08-20

**Authors:** Bradley S. McNeal, Shauna L. Stahlman, Saixia Ying, Symone M. Baker Miller

**Affiliations:** Uniformed Services University of the Health Sciences, Navy Medicine Readiness and Training Command, Bethesda, MD: LCDR McNeal; Armed Forces Health Surveillance Division, Public Health Directorate, Defense Health Agency, Silver Spring, MD: Dr. Stahlman, Dr. Ying, LCDR Baker Miller

## Abstract

This retrospective cohort study evaluated trends in postpartum depression (PPD) and postpartum anxiety (PPA) diagnoses among 138,588 active component service women with live births (2010-2024). Period prevalence of PPD/PPA remained relatively constant from 2010 to 2017 (2.4%) and began increasing in 2018, with a peak in 2022 (13.4%). Multivariable logistic regression (2021–2024) demonstrated a statistically significant decrease in adjusted odds of PPD/PPA diagnosis in 2023-2024 (adjusted odds ratio 0.89), following the pandemic peak. The most significant risk factors for PPD/PPA diagnosis were prior mental health history, service in the Navy, and cesarean delivery. The 15-year increase in diagnostic prevalence may reflect improved administrative documentation and reduced stigma associated with seeking care. Conversely, the post-2022 decline may reflect post-pandemic stabilization and mitigating effects of expanded parental leave. Current PPD/PPA prevalence remains higher than pre-pandemic proportions, however. Prior mental health diagnosis remains the critical predictor, identifying a key population requiring targeted support to enhance military readiness.

Postpartum depression (PPD) and postpartum anxiety (PPA) present significant public health concerns and are the most prevalent complications following childbirth in the U.S. As many as 14% of childbearing individuals experience PPD, and another 6–8% experience PPA. ^1^ In addition to general risks for PPD/PPA experienced by their civilian counterparts, U.S. active component service women (ACSW) face unique stressors from military life, including family separation, frequent relocation, deployments, and combat exposures with pre-existing trauma. ^2^ Career and duty limitations, whether actual or perceived, may preclude ACSW from seeking mental health services and receiving care, ^3^ and duty requirements can inhibit appointment attendance. ^4^ Untreated mental health conditions may result in delays in returning to duty, difficulties in completing necessary tasks upon return to work, and ultimately, may result in subsequent duty limitations or service separations for ACSW.

Pregnancy, childbirth, and parenthood are times of great stress for ACSW, and hormonal shifts occurring from the antenatal to postpartum periods can briefly affect mood. “Baby blues,” a common and normal phenomenon, occurs typically in the first 2-3 days after delivery. Feelings of sadness and lability of mood are not a *Diagnostic and Statistical Manual of Mental Disorders, Fifth Edition, Text Revision* (DSM-5-TR) diagnosis and are not pathological. Management involves non-clinical psychosocial support (e.g., reassurance, familial support, rest), and symptoms typically resolve no later than 2 weeks. ^5 , 6^

Unlike ‘baby blues’, PPD is characterized by greater intensity and persistence, for more than 2 weeks, hindering ability to care for one’s infant and self. ^5 , 6^ Symptoms of PPD include fatigue, irritability, frequent crying, difficulty with infant bonding, prolonged sadness, changes in sleep and appetite, feelings of guilt or worthlessness, and social withdrawal. In severe cases, individuals may experience thoughts of self-harm or suicide. Symptoms can appear within the first few weeks postpartum but may also develop during pregnancy or as long as 1 year after giving birth. ^5 , 6^ Postpartum anxiety may occur independently or concurrently with PPD, with symptoms including excessive worry, restlessness, panic attacks, and heart palpitations. Risk factors for PPD/PPA include personal or family history of mood disorder, hormonal fluctuations, sleep deprivation, lack of social support, relationship stress, and unplanned or complicated pregnancy. ^5^

**What are the new findings?**

Period prevalence of PPD/PPA peaked in 2022 before declining in 2023 and 2024, while remaining higher than pre-pandemic levels. Service in the Navy, prior mood disorders, and cesarean delivery correlated with increased diagnostic odds, while Air Force and Space Force service, as well as officer status, were associated with reduced odds compared to Army and enlisted personnel.

**What is the impact on readiness and force health protection?**

Unrecognized postpartum depression and anxiety detrimentally affect return to duty and overall deployability. The identification of atrisk populations and implementing policies to mitigate the impacts of postpartum mood disorders are critical for sustaining the health, readiness, and retention of service women in the U.S. military.

Systemic changes over the past 15 years could have affected PPD/PPA prevalence. In 2015, the U.S. Department of Defense extended postpartum deployment deferments from 6 to 12 months. In 2016, maternity leave (in addition to 6 weeks medical convalescent leave) for the birth parent increased to 12 weeks ^7^ ; this increase was from 6 weeks in the Army and Air Force; the Navy and Marine Corps had established an 18-week policy in July 2015. In 2018, TRICARE eliminated the primary care referral requirement for routine, outpatient mental health care, which significantly lowered institutional barriers to access. ^8^ Concurrently, in October 2018, a new International Classification of Diseases, 10th Revision, Clinical Modification (ICD-10-CM) code, F53.0, was introduced to provide specificity for PPD. From March 2020 through March 2023, the COVID-19 pandemic affected mental health due to stress and isolation. ^9^ In 2023, parental leave for service members was further expanded, granting secondary caregivers 12 weeks of leave to provide additional support. Lastly, in 2023, the Brandon Act was enacted to reduce stigma and establish command-initiated approaches and access to mental health care, which potentially influenced care-seeking behaviors, although its direct impact on reducing stigma among service women remains difficult to isolate. ^10^

This study aimed to evaluate the period prevalence of PPD/PPA diagnoses among U.S. ACSW from 2010 through 2024, in concurrence with the systemic changes to related policies during the period. As a secondary objective, this analysis examined potential demographic and clinical risk factors for PPD/PPA during the last 4 years of the surveillance period.

## Methods

This retrospective cohort study utilized data from January 1, 2010 through December 31, 2024 from the Defense Medical Surveillance System (DMSS). The study population included all ACSW in the U.S. Army, Navy, Air Force, Space Force, and Marine Corps who had a live birth documented while in active service during the study period. To ensure independent observations and eliminate the compounding clinical effects of multi-parity within administrative data, the study cohort was restricted to an individual’s first documented live birth event during the study period after accession into military service.

Analysis focused on this index inpatient or outpatient encounter with a live birth diagnosis in any diagnostic position (ICD-9-CM: V27.0, V27.2, V27.5, V27.9, V30*, V31*, V33*, V34*, V37*, V39*; ICD-10-CM: Z37.0, Z37.2, Z37.5*, Z37.9, Z38*). Subsequent births by the same individual during the 15-year surveillance period were excluded from the denominator: Each unique individual contributed exactly 1 delivery event and a single 365-day post-partum follow-up period to the analysis; this did not limit the dataset strictly to primiparous women if prior births occurred before the start of military service. This broad diagnostic approach was used to maximize baseline case capture in administrative medical records, and allowed capture of deliveries that may have occurred at home or in civilian health care facilities. Pregnancies resulting in stillbirth were excluded to avoid confounding postpartum trends with normal bereavement.

Based on the timings of policy changes, changes to the ICD-CM coding system, and the COVID-19 pandemic, for analysis the study period was divided into 5 distinct eras: 2010–2015 (baseline), 2016–2018 (ICD-10-CM, post-maternity leave expansion, 2018 TRICARE removal of outpatient mental health referral requirements), 2019–2020 (post-introduction of ICD-10-CM code F53.0, early pandemic), 2021–2022 (late pandemic), and 2023–2024 (post-parental leave expansion, pandemic, Brandon Act).

The primary outcome was a diagnosis of PPD or PPA. Cases were defined as at least 1 inpatient or outpatient medical encounter with a qualifying diagnosis code in any diagnostic position from 14 through 365 days after a live birth; the 2-week exclusion eliminated transient ‘baby blues’ from analysis. ^5 , 6^ The 1-year end date was implemented because prior studies indicate that 57.4% of new diagnoses are found at the 7-10 month follow-up, followed by subsequent decrease in diagnostic prevalence. ^6^ Qualifying diagnoses of PPD/PPA were identified using ICD-9-CM codes 648.42 (mental disorders of mother, delivered, with mention of postpartum complication) and 648.44 (mental disorders of mother, postpartum condition or complication), and ICD-10-CM codes F53.0 (postpartum depression), O90.6 (postpartum mood disturbance), and O99.345 (other mental disorders complicating the puerperium).

Two variations of the outcome were analyzed in a sensitivity analysis to calculate 2 different prevalence estimates, to assess the impact of coding variations on the unadjusted proportions of PPD/PPA. The first outcome variation was identical to the primary definition, except that ICD-9-CM code 648.40 (“mental disorders of the mother, unspecified as to episode of care or not applicable”) was included. Tracking for code 648.40 was only used during the base-line period, from January 1, 2010 through September 30, 2015, as ICD-9-CM transition to ICD-10-CM occurred on October 1, 2015. This sensitivity analysis was included because, although non-specific to postpartum states, code 648.40 was used in previous studies, notably Herrick et al. ^10^ and provides an essential baseline for tracking historical trends.

The second outcome variation in the sensitivity analysis removed the 14-day exclusion period (observing days 0–365) and expanded diagnostic criteria to any anxiety or depressive disorder. This broader outcome definition was tested because providers may utilize non-specific psychiatric codes during the postpartum period, either due to clinical uncertainty or avoidance of administrative stigma.

Demographic variables considered in analysis included age at delivery, race and ethnicity, and marital status. Records with missing or unspecified race or ethnicity data were retained in the analysis and classified as ‘unknown’. Military variables included rank, service branch, and deployment history prior to the index delivery date (which was restricted to earlier than 2022 due to DMSS data limitations).

Clinical covariates included mode of delivery (cesarean vs. vaginal) and prior diagnosis of adjustment disorder, anxiety disorder, or depressive disorder in any diagnostic position of an inpatient or outpatient encounter prior to the delivery date. The ICD-9-CM / ICD-10-CM codes used for depression, anxiety, and adjustment disorder are described in a prior *MSMR* report. ^11^ Secondary analysis used multivariate logistic regression restricted to the contemporary 2021-2024 surveillance period to assess independent associations with PPD/PPA diagnosis. This temporal restriction was applied to prevent confounding from historical ICD-CM coding transitions and pandemic-related access distortions.

While this study is fundamentally descriptive, multivariable logistic regression yielding adjusted odds ratios (aORs) was employed to isolate independent risk factors by controlling for inherent demographic confounding (e.g., collinearity between military rank and age). This methodology remains statistically robust, as the estimates are interpreted as odds ratios rather than risk ratios, which would otherwise require a rare disease assumption. The model adjusted for maternal age, race and ethnicity, service branch, military rank, prior deployment history, mode of delivery, and prior mental health diagnosis. Analysis was performed using SAS Enterprise Guide version 8.4.

## Results

The final study cohort comprised 138,588 first documented live births among ACSW from 2010 through 2024. Detailed baseline demographic, clinical, and military characteristics for the complete cohort are detailed in the “Overall” column rows of **Table 1** . The majority of delivery events occurred among individuals ages 20-29 years (76.9%), of non-Hispanic White race or ethnicity (43.6%), and in enlisted ranks (85.2%). The U.S. Army contributed the largest volume of delivery records (37.9%), followed by the Navy (27.6%), Air Force (26.3%), and Marine Corps (8.2%).

**TABLE 1.** Incidence of Postpartum Depression or Postpartum Anxiety Following Live Birth, Active Component Service Women, U.S. Armed Forces, 2010–2024

Characteristics	Total, 2010–2024		2010–2015		2016–2018		2019–2020		2021–2022		2023–2024	
	No.	%	No.	PP	No.	PP	No.	PP	No.	PP	No.	PP
Any depression or anxiety disorder diagnosis postpartum	138,588	100	9,416	16.7	4,704	17.6	4,202	21.8	5,604	29.4	5,639	32.7
PPD, PPA diagnoses by strict, primary parameters	9,522		1,331	2.4	1,370	5.1	2,135	11.1	2,551	13.4	2,135	12.4
ICD-9-CM 648.40 included ^a^			2,922	5.2	—	—	—	—	—	—	—	—
Age, *y*												
<20	3,980	2.9	60	3.5	45	5.7	82	13.6	71	15.1	38	9.0
20–24	65,342	47.2	692	2.5	720	5.7	1,103	12.2	1,254	14.5	953	13.1
25–29	41,163	29.7	355	2.1	372	4.8	619	10.7	774	13.4	670	12.6
30–34	20,314	14.7	163	2.1	158	4.1	249	9.0	339	11.3	339	11.2
35+	7,789	5.6	61	2.2	75	4.7	82	7.7	113	9.7	135	11.2
Race and ethnicity												
White, non-Hispanic	60,367	43.6	666	2.6	630	5.5	928	11.6	1,027	13.1	822	11.8
Black, non-Hispanic	33,630	24.3	324	2.3	331	4.8	542	11.5	640	14.4	474	12.7
Hispanic	26,608	19.2	213	2.4	226	4.6	427	10.4	580	13.2	571	13.0
Other	15,407	11.1	110	1.7	156	5.2	198	9.9	248	12.3	240	12.6
Unknown	2,576	1.9	18	1.7	27	5.1	40	9.5	56	15.3	28	12.7
Branch of service												
Army	52,449	37.9	804	3.6	546	5.4	858	11.9	951	13.6	748	12.4
Navy	38,308	27.6	253	1.7	380	5.0	580	10.9	835	15.1	712	14.3
Air Force, Space Force	36,475	26.3	184	1.3	312	4.6	525	10.0	585	11.3	519	10.8
Marine Corps	11,356	8.2	90	1.9	132	5.9	172	11.2	180	13.2	156	10.8
Rank												
Junior enlisted (E1–E4)	80,682	58.2	956	2.8	895	5.7	1,356	12.2	1,564	14.9	1,128	12.8
Senior enlisted (E5–E9)	37,345	27.0	263	1.9	332	4.8	588	10.9	747	13.2	724	13.3
Warrant officer (W0)	384	0.3	2	1.2	5	7.1	3	7.1	7	13.7	5 9.6
Junior officer (O1–O3)	16,032	11.6	91	1.5	117	3.6	158	7.4	194	8.9	222	9.8
Senior officer (O4–O10)	4,145	3.0	19	1.3	21	2.6	30	4.9	39	6.4	56	8.6
Military occupation												
Combat-related	3, 727	2.7	26	2.4	29	4.4	60	9.9	92	12.9	71	10.4
Motor transport	5,331	3.9	37	1.7	59	5.6	83	11.0	102	13.6	80	12.3
Pilot, air crew	1,821	1.3	5	0.7	9	2.6	15	5.8	21	8.1	21	7.2
Repair, engineering	30,049	21.7	274	2.2	306	5.0	439	10.8	574	14.5	450	13.3
Communications, intelligence	45,760	33.0	491	2.6	452	5.3	711	11.2	807	13.2	704	12.7
Health care	25,531	18.4	251	2.4	261	5.4	413	11.6	436	12.4	397	12.7
Other	26,369	19.0	247	2.4	254	5.0	414	11.3	519	13.9	412	11.5
Marital status												
Single, never married	33,384	24.1	318	2.2	318	5.5	555	12.2	648	14.2	543	12.5
Married	97,933	70.7	929	2.4	991	5.0	1,432	10.5	1,749	13.1	1,449	12.3
Other	7,238	5.2	84	3.2	61	4.7	148	13.4	154	13.4	143	13.3
Unknown	33	0.0	—	—	—	—	—	—	—	—	—	—
Delivery type												
Vaginal	111,222	80.3	1,024	2.3	1,051	4.8	1,763	11.0	2,055	12.9	1,598	12.2
Cesarean section	27,366	19.8	307	2.6	319	6.4	372	11.5	496	15.9	537	12.8
Prior depressive disorder diagnosis												
Yes	20,511	14.8	402	5.0	306	9.0	469	18.4	574	18.7	574	17.0
No	118,077	85.2	929	1.9	1,064	4.6	1,666	10.0	1,977	12.4	1,561	11.3
Prior anxiety disorder diagnosis												
Yes	22,442	16.2	292	4.3	345	8.6	575	17.8	706	18.2	754	16.4
No	116,146	83.8	1,039	2.1	1,025	4.5	1,560	9.7	1,845	12.2	1,381	10.9
Prior adjustment disorder diagnosis												
Yes	32,425	23.4	479	4.2	456	7.6	751	15.9	900	17.5	823	16.0
No	106,163	76.6	852	1.9	914	4.4	1,384	9.5	1,651	11.9	1,312	10.9
Prior deployment (prior to 2022)												
Never deployed	94,829	68.4	826	2.6	998	5.4	1,667	11.6	2,203	13.8	1,807	12.6
Deployed, 1+	43,759	31.6	505	2.1	372	4.5	468	9.6	348	11.2	328	11.1

Abbreviations: No., number; PP, period prevalence; PPD, postpartum depression; PPA, postpartum anxiety; ICD-9-CM, International Classification of Diseases, 9th Revision, Clinical Modification; *y* , years.

a Includes ICD-9-CM code 648.40 strictly for the period January 1, 2010–September 30, 2015; following October 1, 2015 system-wide transition to ICD-10-CM, this historical variation corresponds identically with primary case definition due to retirement of 648.40 indicator.

Utilizing the primary strict case parameters, a total of 9,522 unique PPD/PPA conditions were identified throughout the entire surveillance period. From 2010 to 2015, the period prevalence of PPD/PPA was 2.4% using the primary outcome definition, which increased to 5.2% when ICD-9-CM code 648.40 was included. The proportion of depressive or anxiety disorder diagnoses during the 2010-2015 period was 16.7%. The period prevalence of PPD/PPA, using the primary outcome definition, increased from 5.1% in 2016–2018 to 11.1% in 2019-2020. In 2021-2022, the upward trend continued, with period prevalence peaking at 13.4%, before falling to 12.4% in 2023-2024. The proportion of depressive or anxiety disorder diagnoses also increased from 17.6% in 2016–2018 to 21.8% in 2019-2020, 29.4% in 2021-2022, and continued to increase to 32.7% in 2023-2024 **(Table 1** , **Figure)** .

**FIGURE:**
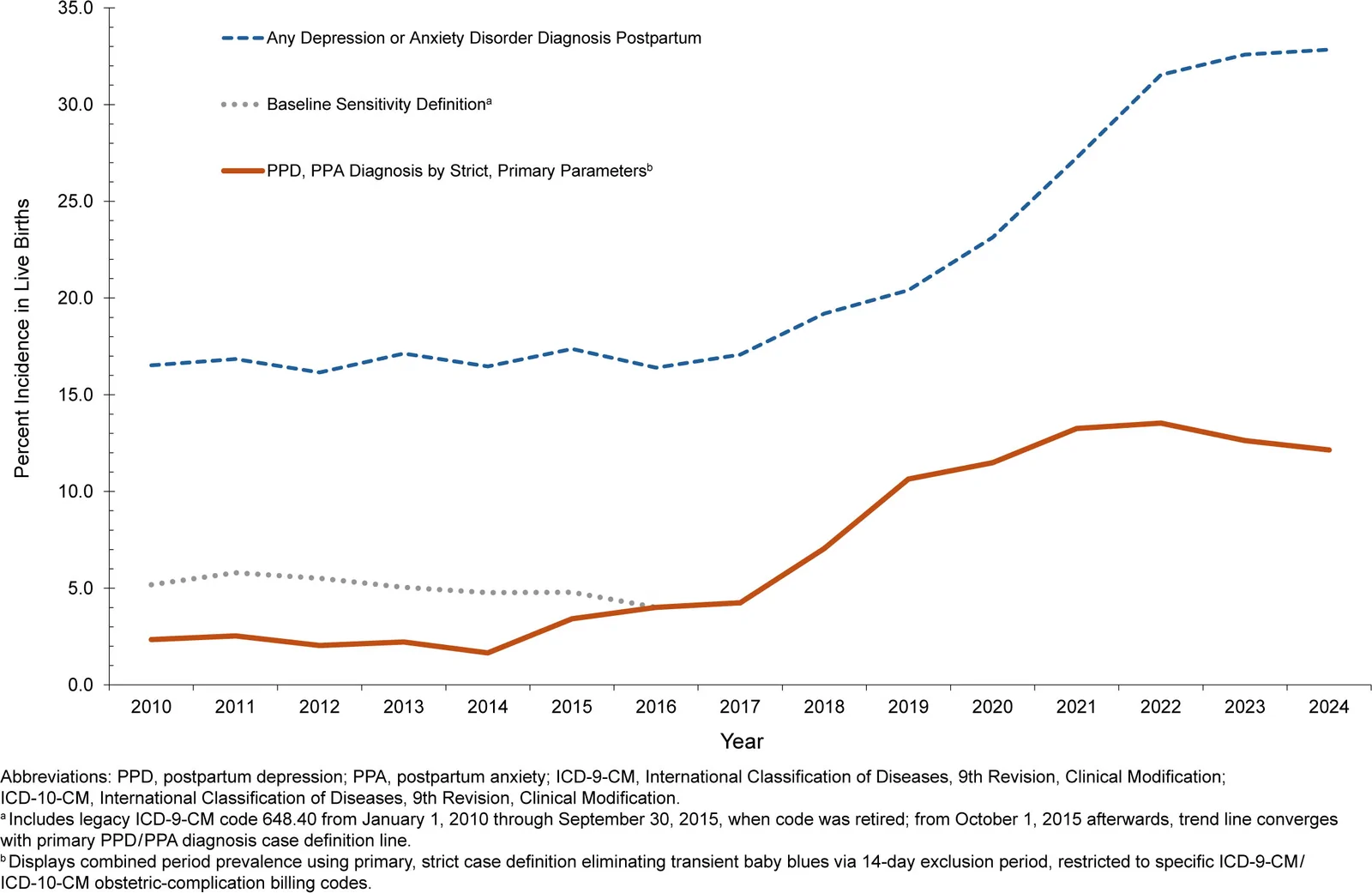
Trends of Postpartum Depression and Postpartum Anxiety Proportions by Case Definition Variation, Active Component Service Women, U.S. Armed Forces, 2010–2024

There was significant decrease in odds of PPD/PPA diagnosis from the 2021-2022 to 2023-2024 periods (aOR 0.89, *p* =0.0001) **(Table 2)** . Compared to soldiers in the Army, Navy sailors exhibited a statistically significant 15% increase in odds of postpartum mood diagnosis (aOR 1.15, *p* <0.001), while Air Force and Space Force members demonstrated a statistically significant 13% reduction in odds (aOR 0.87, *p* =0.002). Compared to junior enlisted members, junior and warrant officers (aOR 0.73, *p* <0.0001), as well as senior officers (aOR 0.60, *p* <0.0001) had reduced odds of diagnosis. Delivery by cesarean section was associated with increased odds of diagnosis (aOR 1.16, *p* <0.0001), as was prior depression (aOR 1.22, *p* <0.0001), anxiety (aOR 1.33, *p* <0.0001), and adjustment disorder (aOR 1.29, *p* <0.0001). History of deployment prior to the index childbirth was not a statistically significant independent predictor of diagnosis in the adjusted model (aOR 0.92, *p* =0.074).

**TABLE 2.** Multivariable Logistic Regression Analysis of Demographic and Clinical Risk Factors Associated with Primary, Specific Postpartum Depression or Postpartum Anxiety Criteria, Active Component Service Women, U.S. Armed Forces, 2021–2024

Characteristics	Unadjusted				Adjusted ^a^			
	Odds Ratio	95% LL	95% UL	*p* -value	Odds Ratio	95% LL	95% UL	*p* -value
Period of time								
2021–2022	Reference				Reference			
2023–2024	0.91	0.86	0.97	0.0040	0.89	0.83	0.94	0.0001
Age, *y*								
<20	0.87	0.70	1.06	0.1659	0.92	0.75	1.13	0.4093
20–24	Reference				Reference			
25–29	0.93	0.86	1.00	0.0388	0.97	0.90	1.05	0.4909
30–34	0.79	0.72	0.86	<0.0001	0.91	0.81	1.01	0.0741
35+	0.73	0.63	0.83	<0.0001	0.85	0.72	1.00	0.0565
Race and ethnicity								
White, non-Hispanic	Reference				Reference			
Black, non-Hispanic	1.10	1.02	1.20	0.0155	1.01	0.92	1.09	0.9045
Hispanic	1.06	0.98	1.15	0.1584	1.01	0.93	1.09	0.9080
Other	1.00	0.90	1.11	0.9496	0.99	0.89	1.10	0.7990
Unknown	1.17	0.92	1.48	0.1914	1.11	0.87	1.41	0.3901
Branch of service								
Army	Reference				Reference			
Navy	1.15	1.07	1.24	0.0002	1.15	1.07	1.25	0.0004
Air Force, Space Force	0.83	0.77	0.90	<0.0001	0.87	0.80	0.95	0.0020
Marine Corps	0.90	0.80	1.02	0.1058	0.89	0.78	1.01	0.0667
Rank								
Junior enlisted (E1–E4)	Reference				Reference			
Senior enlisted (E5–E9)	0.95	0.88	1.01	0.1022	0.91	0.84	0.99	0.0292
Junior officer (O1–O3, WO1–WO3)	0.64	0.58	0.72	<0.0001	0.73	0.64	0.83	<0.0001
Senior officer (O4–O10, W04–WO5)	0.50	0.41	0.62	<0.0001	0.60	0.47	0.77	<0.0001
Military occupation								
Combat-related	Reference				Reference			
Motor transport	1.13	0.90	1.41	0.2972	1.03	0.82	1.30	0.7998
Pilot, air crew	0.62	0.44	0.89	0.0087	0.94	0.65	1.34	0.7148
Repair, engineering	1.22	1.02	1.45	0.0280	1.13	0.94	1.35	0.1998
Communications, intelligence	1.13	0.95	1.34	0.1798	1.12	0.94	1.33	0.2250
Health care	1.08	0.90	1.29	0.3931	1.07	0.89	1.28	0.5031
Other	1.10	0.92	1.32	0.2768	1.14	0.95	1.37	0.1597
Marital status								
Single, never married	Reference				Reference			
Married	0.94	0.88	1.01	0.1165	1.03	0.96	1.11	0.4469
Other, unknown	0.99	0.87	1.14	0.8989	0.97	0.84	1.11	0.6449
Delivery type								
Vaginal	Reference				Reference			
Cesarean section	1.14	1.06	1.23	0.0005	1.16	1.08	1.25	0.0001
Prior depressive disorder diagnosis								
Yes	1.61	1.50	1.73	<0.0001	1.22	1.12	1.33	<0.0001
No	Reference				Reference			
Prior anxiety disorder diagnosis								
Yes	1.59	1.48	1.70	<0.0001	1.33	1.22	1.44	<0.0001
No	Reference				Reference			
Prior adjustment disorder diagnosis								
Yes	1.56	1.46	1.66	<0.0001	1.29	1.20	1.39	<0.0001
No	Reference				Reference			
Prior deployment (prior to 2022)								
Never deployed	Reference				Reference			
Deployed, 1+	0.82	0.75	0.89	<0.0001	0.92	0.83	1.01	0.0743

Abbreviations: LL, lower limit; UL, upper limit; *y* , years; PPD, postpartum depression; PPA, postpartum anxiety; ICD-9-CM, International Classification of Diseases, 9th Revision, Clinical Modification; ICD-10-CM, International Classification of Diseases, 10th Revision, Clinical Modification.

a Multivariable model adjusted for all listed covariates concurrently; complete covariate adjustment set includes maternal age, race or ethnicity, service branch, and military rank, pay grade. Outcome restricted exclusively to the primary, specific PPD/PPA case definition (ICD-9-CM: 648.42, 648.44; ICD-10-CM: F53.0, O90.6, O99.345). High prevalence, non-specific sensitivity outcome variations were excluded from multivariable modeling.

## Discussion

Over the 15-year period, diagnoses of PPD and PPA increased overall among ACSW, with substantial increases in diagnoses from 2018, and through most of the pandemic, peaking in 2022. Introduction of the ICD-10-CM code F53.0 in 2018 may have influenced PPD case identification and surveillance, thereby improving diagnosis and data capture. Before code F53.0, there were multiple non-specific diagnostic codes, which makes it challenging to compare recent trends with the years preceding 2019. Furthermore, the massive gap between this study’s strict primary outcome criteria (2.4-13.4%) and the broader sensitivity criteria (16.7-32.7%) highlights the potentially wide variability in prevalence estimates of PPD, depending on which ICD-10-CM codes are selected. Strict definitions maximize specificity but inevitably undercount total distress due to provider coding omissions or hesitance to apply formal postpartum diagnoses. Conversely, the broader definition maximizes sensitivity to capture logistical demand but introduces misclassification bias by sweeping in chronic, pre-existing, or unrelated psychiatric conditions that coincidentally occur during the postpartum year. Using both definitions provides health analysts with the operational boundaries between true obstetric complications and overall behavioral health demand.

The peak in period prevalence of PPD/PPA in 2022 correlates with increased diagnoses of several mental health disorders from 2021 through 2023 reported in the December 2024 issue of *MSMR* . ^11^ Specifically, that report documented significant force-wide increases in the incidence of adjustment disorders, anxiety disorders, and depressive disorders from 2021 to 2023 within the U.S. active component, highlighting that crude incidence rates (IRs) for these specific mental health categories were consistently and substantially higher among service women compared to service men throughout the surveillance period. ^11^ The rise in PPD/PPA diagnoses in this study cohort is, therefore, not isolated but correlates with broader trends in mental health diagnoses. Period prevalence of PPD/PPA decreased significantly in the 2023-2024 period compared to 2021-2022, however, which differed from diagnoses of depressive and anxiety disorder that continued to increase in 2023-2024.

The primary strict postpartum depression period prevalence (2.4%) in this study is consistent with, although slightly lower than, the peak administrative IR of approximately 3.0% reported by Nicholson et al. ^12^ This variation is methodologically expected, as our model implemented a strict 14-day postpartum exclusion period to systematically eliminate transient, early behavioral health encounters. Conversely, the higher overall psychiatric care proportions reported by Moore et al. (12.5% within 1 year postpartum) and assessed globally in multi-year Military Health System profiles (e.g., Manzo et al.) ^13 , 14^ are explained by broader operational case parameters. While this study’s primary models captured only encounters explicitly coded with specific obstetric or pregnancy-complication billing markers, the other study cohorts assessed a comprehensive array of general depressive, anxiety, and trauma-related disorders. In this study’s secondary sensitivity analysis, which removed the exclusion period and included general psychiatric billing codes, the observed rate of 16.7% correlated with those broader, force-wide surveillance studies, demonstrating a precise boundary between localized obstetric pathology and overall medical demand among childbearing service women.

These longitudinal diagnostic trends are temporally coincident with major systemic and environmental changes, including the expansion of parental leave policies, initiatives to reduce mental health stigma, such as the Brandon Act, and widespread adoption of telehealth. Notably, this study’s observations align with prior military cohorts: For example, Herrick et al. similarly observed a trend of decreasing diagnostic prevalence after revised 2016–2017 parental leave policies, followed by a sharp increase in 2018 and 2019. ^10^

As a descriptive study, this analysis cannot determine causality nor isolate the direct impacts of those systemic changes. This study cannot definitively state whether the rising diagnostic trends reflect a true increase in the underlying population prevalence of these disorders or simply an increase in health care provision due to lowered structural barriers to care. For instance, while expanded parental leave is generally associated with improved maternal mental health, it simultaneously provides service women with the necessary time and flexibility to seek medical care, which could, theoretically, increase documented administrative diagnoses. The 2023-2024 decline in PPD/PPA period prevalence suggests that post-pandemic stabilization may have alleviated parental stress, although prevalence remains higher than pre-pandemic levels. This persistent elevation suggests historical under-estimation of pre-pandemic proportions, a new baseline with improved case capture via specific ICD-10-CM coding, a true increase in the proportion of diagnoses, or a combination of these factors. Future analytical studies examining these proportions in 2025 and beyond may provide more insight into this trend.

This analysis demonstrates that ACSW share several risk factors with their civilian counterparts, including increased odds of PPD/PPA diagnosis after a cesarean delivery or with a history of mood disorder. ^15 , 16^ Notably, while unadjusted baseline profiles suggested lower diagnostic prevalence among previously deployed personnel, multivariable modeling demonstrates that prior deployment exerts no independent protective or hazardous association with postpartum outcomes, after accounting for underlying rank and age.

The statistically significant, divergent odds between branches of military service may reflect unique occupational structures, variations in localized care delivery systems, or socio-economic factors. A 15% relative increase in odds for Navy personnel represents a meaningful impact in absolute health care provision and total force medical readiness for a large population. Navy personnel may experience distinct, cluster-level stressors due to isolation at sea, high tempos and erratic schedules, and unique station cultures.

Rather than reflecting a clinical screening bias, these variations among service branches highlight the need for future targeted research into how unique naval operational environments, such as prolonged shipboard deployments, isolated maritime platforms, and localized medical resource constraints, can affect maternal mental health and subsequent provision of care. Decreased odds of diagnosis among officers compared to enlisted personnel may be due to greater age and life experience, along with lower stress and higher income. Additionally, there is documented relative aversion among senior leaders and officers to present for behavioral health diagnoses; prior military health research indicates that this population faces unique attitudinal barriers to care, including heightened concerns about professional reputation, career longevity, and an internalized sense of obligation to maintain continuous departmental performance. ^17^

This study has several limitations, including its use of historical data and codes that complicates capture of diagnoses not definitively coded in the medical record. Crucially, lack of a dedicated, specific PPA diagnostic code in the ICD-CM system necessitates that providers utilize generic anxiety codes, which inhibits isolation of a reliable metric for concurrent, comorbid PPD/PPA in maternal surveillance data. Furthermore, this study could not account for variation in symptom severity or treatment modality. Because this study relied solely on administrative medical encounter data to identify live births, without independent validation through personnel registries, there is inherent risk of denominator misclassification. In addition, it was not possible to account for births that preceded military service. This study could not determine whether service members used parental leave immediately following convalescence or distributed it throughout the ensuing year. Furthermore, this observational design prevents establishment of causality between policy changes and period prevalence.

Moreover, these findings likely underestimate true PPD/PPA prevalence, as some service members may not seek care or utilize short-term, non-medical counseling services (i.e., chaplains, military family and life counseling) that do not generate medical diagnoses. Data were unavailable for deployments after 2022. Additionally, early separation from military service following childbirth inherently limits long-term medical accounting for those specific individuals within DMSS. Finally, unmeasured confounders, such as personal or operational stress, along with social support systems, may influence service member stress and could not be accounted for in this study.

Future research could disentangle the effects of concomitant initiatives by isolating confounding factors and events identified by this study, to better understand which interventions are most effective. Qualitative studies could assess the lived experiences of service members in the identified periods and effects of specific events, for better insights into how policy changes, family-focused initiatives, the pandemic, and efforts to reduce stigma have influenced the experiences of postpartum service members. Further studies are needed to evaluate whether policy decisions and MHS provision of care are adequately addressing the needs of service members, for maintaining a ready fighting force.
